# Persistent predominance of the Victoria lineage of influenza B virus during COVID‐19 epidemic in Nanchang, China

**DOI:** 10.1111/irv.13226

**Published:** 2023-12-10

**Authors:** Xianfeng Zhou, Ziqi Lin, Junling Tu, Chunlong Zhu, Hui Li

**Affiliations:** ^1^ Jiangxi University of Chinese Medicine Nanchang China; ^2^ Nanchang Center for Disease Control and Prevention Nanchang China; ^3^ Third Hospital of Nanchang Nanchang China

**Keywords:** COVID‐19, influenza B, influenza‐like illness, pathogen spectrum, Victoria lineage

## Abstract

The sentinel hospital‐based influenza‐like illness (ILI) surveillance network was established in China since the 2009 H1N1 pandemic. This network plays important roles in monitoring influenza virus variation and identifying novel respiratory pathogens. In this study, we characterized the pathogen spectrum pattern (PSP) of ILI based on three sentinel hospitals and analyzed the significant change of PSP during the COVID‐19 epidemic. The notable change of influenza virus spectrum was observed since the beginning of COVID‐19 outbreak, and we found persistent domination of Victoria lineage of influenza B virus and “extinction” of A/H1N1, A/H3N2, and B/Yamagata during the dynamic Zero‐COVID‐19 pandemic in Nanchang, China. However, these strains intermittently co‐circulated before the COVID‐19.

The influenza B virus belongs to the family Orthomyxoviridae. Two antigenically and genetically distinct lineages Victoria (B/Victoria) and Yamagata (B/Yamagata) were classified since 1980s.^1^ It has been proposed that the differences in the evolutionary dynamics of these two lineages are due to the binding preferences of hemagglutinin to the cellular receptor. The B/Victoria is able to bind to cell receptors with sialic acid residues at the α‐2,3 and α‐2,6 positions, whereas the B/Yamagata exclusively bind to residue at the α‐2,6 positions of the respiratory tract.^1^ Since the outbreaks of COVID‐19, the “extinction” of B/Yamagata lasted for over 2 years around the world according to the database of Nextstrain. However, the B/Victoria predominated and evolved into the major clade V1A.3a.2. It is essential to answer the following questions: (1) What caused this evolutionary polarization of B/Victoria? (2) Does the anti‐COVID‐19 pandemic measures preferably restrain the transmission of B/Yamagata and other seasonal flu?

Here, we retrospectively analyzed the weekly pathogen spectrum of influenza viruses based on influenza‐like illness (ILI) surveillance from three local sentinel hospitals in the southeastern Chinese city of Nanchang, including a provincial children's hospital during 2014–2022. The national ILI surveillance network was established since the H1N1 pandemic in 2009.^2^ During the past 14‐year surveillance, the network played an important role in identifying novel pathogens from humans such as H7N9 in 2013, H10N8 in 2014, H5N6 in 2014, and H3N8 in 2022.^2^, ^3^, ^4^, ^5^ Since the local H7N9‐ and H10N8‐associated cases were reported during 2013–3014, multiple measures including temporary shutdown of wild‐bird markets and enhanced monthly monitoring were implemented in Nanchang, China.^6^, ^7^ After the outbreak of COVID‐19, the ILI surveillance network also functioned to monitor SARS‐Cov‐2 for all ILI cases from the local sentinel hospitals.

Generally, 30–40 ILI samples were collected from each sentinel hospital per week and sent to Nanchang Center for Disease Control and Prevention (CDC) for molecular surveillance of influenza viruses. Specifically, the ILI cases were diagnosed by clinicians according to diagnostic criteria: fever (body temperature greater than or equal to 38°C), with cough or sore throat, and lack of other laboratory diagnostic evidence. Then the nasopharyngeal swabs from the ILI cases were collected and sent to Nanchang CDC for real‐time RT‐PCR targeting H1N1, H3N2, B/Victoria, and B/Yamagata using commercial kits (DAAN Gene, Guangzhou).

Weekly distribution of influenza‐positive cases in three sentinel hospitals suggested a good consistency of influenza prevalence by ILI surveillance, reflecting the trends of influenza epidemics in the region during 2014–2022 (Figure 1A). As surveillance data indicated in Figure 1B, A/H1N1 mainly prevailed around December of 2014, 2018, and 2019, A/H3N2 mainly prevailed from May to October during 2014–2017. During 2016–2018, A/H1N1, A/H3N2, and FluB co‐circulated. The local “extinction” of B/Yamagata occurred since the end of 2018 (Figure 1B,C) and “extinction” of A/H1N1pdm and A/H3N2 was also observed from May 2020 to March 2022 when the B/Victoria monopolized till the subsequent emersion of A/H3N2 peaking around April 2022 (Figure 1B,C). Weekly distribution of each type of influenza virus indicated typical seasonality of H1N1 prevailing in the first 12 weeks, while H3N2 in 20–32 weeks. However, FluB maintained similar prevalence levels throughout the year (Figure 1D). When multiple pathogens co‐circulate, this can lead to competitive or cooperative forms of pathogen–pathogen interactions. It is believed that such interactions occur among influenza viruses, perhaps through broad‐acting immunity, resulting in interlinked epidemiological patterns of infection.^8^ The local “extinction” of A/H1N1, B/Yamagata, and A/H3N2 since the COVID outbreak in early 2020 might be associated with negative interaction of SARS‐CoV‐2 to these influenza viruses. The COVID‐19 pandemic and subsequent implementation of nonpharmaceutical interventions (NPIs) (e.g., cessation of global travel, wearing mask, physical distancing, and staying home) reduced transmission of some viral respiratory pathogens, most notably pediatric respiratory syncytial virus (RSV) and influenza.^9^ To our knowledge, A/H1N1, A/H3N2, and B/Yamagata tend to bind to cell receptors with human sialic acid residues at the α‐2,6 positions, while B/Victoria is capable to bind to cell receptors with sialic acid residues at the α‐2,3 and α‐2,6 positions.^1^ Will dual‐receptor binding make B/Victoria transmission more competitive than other strains during COVID‐19 pandemic under strict NPIs remain unknown, while our data indicated that the NPIs of COVID‐19 might play a key role in curbing transmission of other respiratory diseases and even fecal–oral transmitted diseases such HFMD (unpublished data).

**FIGURE 1:**
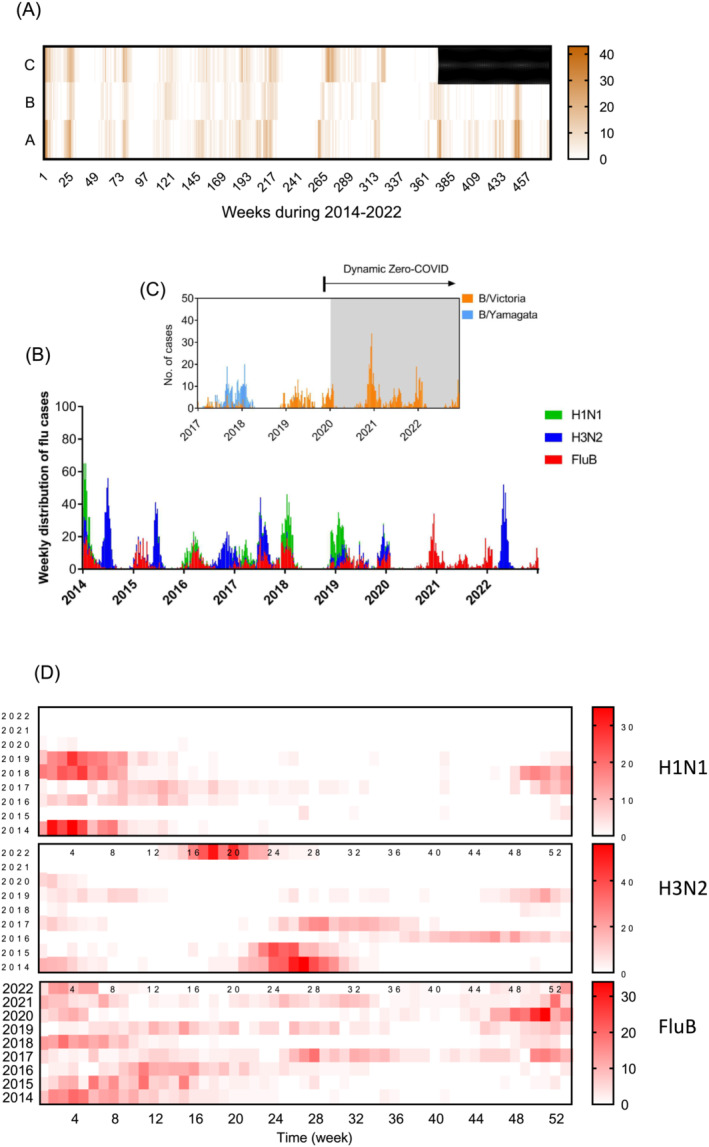
Weekly distribution of influenza viruses in Nanchang, China during 2014–2022. (A) ILI surveillance in three sentinel hospitals: A. hospital A, maternal and child health hospital; B. hospital B, a children's hospital; C. hospital C, a comprehensive tertiary A hospital (the black vacant part indicates that this hospital was requisitioned as a designated treatment hospital for COVID‐19 patients). (B) Weekly distribution of flu cases. (C) weekly cases of B/Victoria and B/Yamagata. (D) Weekly distribution of H1N1, H3N2, and fluB during 2014–2022.

This study reveals the COVID‐19 intervention substantially interfere the pathogen spectrum of the co‐circulating influenza viruses in Nanchang, China. Our study could provide epidemiologically meaningful data for public health. As China shifts its epidemic response policies to manage COVID‐19 from class A to class B infectious diseases, continuous ILI surveillance will be needed to monitor the seasonality pattern of pathogen spectrum and then to provide further insight into possible improvements in the timing of influenza vaccination in Nanchang, China. In the future, the prevention and control of influenza epidemics can learn from the measures of NPIs preventing COVID‐19. And molecular epidemiology of influenza viruses is needed to identify the key mutation on their antigens.

## AUTHOR CONTRIBUTIONS

Xianfeng Zhou and Hui Li conceived and designed the study. Xianfeng Zhou, Junling Tu, and Chunlong Zhu performed experiments. Xianfeng Zhou, Ziqi Lin, and Chunlong Zhu collected data. Xianfeng Zhou, Ziqi Lin, Junling Tu, and Hui Li analyzed and interpreted the data. Xianfeng Zhou and Hui Li wrote the manuscript. All authors approved the manuscript.

## CONFLICT OF INTEREST STATEMENT

None.

### PEER REVIEW

The peer review history for this article is available at https://www.webofscience.com/api/gateway/wos/peer-review/10.1111/irv.13226.

## Data Availability

Data are available on request from the authors.
